# Clinical factors associated with shorter durable response, and patterns of acquired resistance to first-line pembrolizumab monotherapy in PD-L1-positive non-small-cell lung cancer patients: a retrospective multicenter study

**DOI:** 10.1186/s12885-021-08048-4

**Published:** 2021-04-01

**Authors:** Kazutaka Hosoya, Daichi Fujimoto, Takeshi Morimoto, Toru Kumagai, Akihiro Tamiya, Yoshihiko Taniguchi, Toshihide Yokoyama, Tadashi Ishida, Hirotaka Matsumoto, Katsuya Hirano, Ryota Kominami, Keisuke Tomii, Hidekazu Suzuki, Tomonori Hirashima, Satoshi Tanaka, Junji Uchida, Mitsunori Morita, Masaki Kanazu, Masahide Mori, Kenji Nagata, Ikue Fukuda, Motohiro Tamiya

**Affiliations:** 1 Department of Respiratory Medicine, Kobe City Medical Center General Hospital, 2-1-1 Minatojimaminamimachi, Chuo-ku, Kobe-shi, Hyogo 650-0047 Japan; 2 Internal Medicine III, Wakayama Medical University, 811-1, Kimiidera, Wakayama City, Wakayama, 641-8509 Japan; 3 Clinical Research Center, Kobe City Medical Center General Hospital, 2-1-1 Minatojimaminamimachi, Chuo-ku, Kobe-shi, Hyogo 650-0047 Japan; 4 Department of Clinical Epidemiology, Hyogo College of Medicine, 1-1 Mukogawa, Nishinomiya, Hyogo 663-8501 Japan; 5 Department of Thoracic Oncology, Osaka International Cancer Institute, 3-1-69 Otemae, Chuo-ku, Osaka, 541-8567 Japan; 6 Department of Internal Medicine, National Hospital Organization Kinki-Chuo Chest Medical Center, 1180, Nagasone-cho, Kita-ku, Sakai-shi, Osaka, 591-8555 Japan; 7 Department of Respiratory Medicine, Kurashiki Central Hospital, 1-1-1, Miwa, Kurashiki-shi, Okayama, 710-8602 Japan; 8 Department of Respiratory Medicine, Hyogo Prefectural Amagasaki General Medical Center, 2-17-77, Higashi-Naniwa-Cho, Amagasaki-shi, Hyogo 660-8550 Japan; 9 Department of Respiratory Medicine, Himeji Medical Center, 68, Honmachi, Himeji-shi, Hyogo 670-8520 Japan; 10 Department of Thoracic Oncology, Osaka Habikino Medical Center, 3-7-1, Habikino, Habikino-shi, Osaka, 583-8588 Japan; 11 Department of Respiratory Medicine, Osaka General Medical Center, 3-1-56, Bandai-Higashi, Sumiyoshi-ku, Osaka-shi, Osaka, 558-8558 Japan; 12 Department of Respiratory Medicine, Kobe City Medical Center West Hospital, 2-4, Ichiban-cho, Nagata-ku, Kobe-shi, Hyogo 653-0013 Japan; 13 Department of Thoracic Oncology, National Hospital Organization Osaka Toneyama Medical Center, 5-1-1, Toneyama, Toyonaka-shi, Osaka, 560-0045 Japan; 14 Department of Respiratory Medicine, Itami City Hospital, 1-100, Koyaike, Itami-shi, Hyogo 664-8540 Japan

**Keywords:** Non-small cell lung cancer, Immunotherapy, Pembrolizumab, Acquired resistance, Oligoprogression

## Abstract

**Background:**

Despite the wide-spread use of immune checkpoint inhibitors (ICIs) in cancer chemotherapy, reports on patients developing acquired resistance (AR) to ICI therapy are scarce. Therefore, we first investigated the characteristics associated with shorter durable responses of ICI treatment and revealed the clinical patterns of AR and prognosis of the patients involved.

**Methods:**

We conducted a retrospective multi-center cohort study that included NSCLC patients with PD-L1 tumor proportion scores of ≥50% who received first-line pembrolizumab and showed response to the therapy. Among patients showing response, progression-free survival (PFS) was investigated based on different clinically relevant factors. AR was defined as disease progression after partial or complete response based on Response Evaluation Criteria in Solid Tumors. Among patients with AR, patterns of AR and post-progression survival (PPS) were investigated. Oligoprogression was defined as disease progression in up to 5 individual progressive lesions.

**Results:**

Among 174 patients who received first-line pembrolizumab, 88 showed response and were included in the study. Among these patients, 46 (52%) developed AR. Patients with old age, poor performance status (PS), at least 3 metastatic organs, or bone metastasis showed significantly shorter PFS. Among 46 patients with AR, 32 (70%) developed AR as oligoprogression and showed significantly longer PPS than those with non-oligoprogressive AR.

**Conclusions:**

Patients with old age, poor PS, at least 3 metastatic organs, or bone metastasis showed shorter durable responses to pembrolizumab monotherapy. Oligoprogressive AR was relatively common and associated with better prognosis. Further research is required to develop optimal approaches for the treatment of these patients.

**Supplementary Information:**

The online version contains supplementary material available at 10.1186/s12885-021-08048-4.

## Background

Lung cancer is the leading cause of cancer-related deaths worldwide [1]. Non-small cell lung cancer (NSCLC) accounts for approximately 80% of all lung cancer cases, and the majority of these are diagnosed at an advanced stage [2, 3]. Recently, immune checkpoint inhibitors (ICIs) have been established as a therapy regimen for several types of malignancies, including advanced NSCLC.

Pembrolizumab, a fully humanized monoclonal anti-programmed cell death 1 (PD-1) antibody, showed better treatment outcomes than platinum-based chemotherapy for previously untreated advanced NSCLC with positive programmed cell death ligand 1 (PD-L1) status [4, 5]. In particular, especially better treatment outcomes were observed for patients with PD-L1 tumor proportion score (TPS) ≥50%. Hence, pembrolizumab monotherapy has become a standard first-line treatment, particularly for patients with PD-L1 TPS of ≥50%.

In case of patients with NSCLC, ICI therapy has shown more durable responses than the existing cytotoxic agents [4–9]. In an earlier study, approximately 40% of patients with previously treated NSCLC having best overall response (BOR) of partial response (PR) or complete response (CR) to PD-1 axis inhibitor therapy showed sustained response after follow-up of at least 2 years [10]. However, despite durable response to PD-1 axis inhibitors, most patients show acquired resistance (AR). Therefore, there has been increasing attention on AR to improve the clinical outcomes of patients receiving PD-1 axis inhibitors. Nevertheless, there are few reports on the clinical features of AR to ICI therapy [11]. Understanding these clinical features is important in facilitating the appropriate treatment strategy for patients with AR.

The aim of our study was to characterize the clinical factors associated with shorter durable responses to ICI therapy and investigate the clinical patterns and prognosis of patients with AR to improve treatment strategy using ICIs.

## Methods

### Study population

We conducted a retrospective cohort study including patients with advanced NSCLC (unresectable stage IIIB or IV disease based on the 7th edition of TNM classification, excluding postoperative recurrence) with PD-L1 TPS of ≥50%, who received pembrolizumab as a first-line therapy between February 1, 2017 and April 31, 2018, and had initial response to it at any of the 11 participating institutions belonging to Hanshin Oncology clinical Problem Evaluation (HOPE) group. We censored the observation on July 31, 2019. The study protocol was approved by the review board of each institution and is registered with UMIN (University Hospital Medical Information Network Clinical Trials Registry of Japan; number 000032470).

In all the patients, the Eastern Cooperative Oncology Group (ECOG) performance status (PS) was evaluated just before the commencement of pembrolizumab therapy. PD-L1 expression was evaluated by immunohistochemical staining using the commercially available PD-L1 IHC 22C3 pharmDx assay (Dako North America). The time between the date of pembrolizumab commencement and that of disease progression/death (progression-free survival or PFS) or death alone (overall survival or OS) was calculated for each patient. Tumor responses were assessed according to the Response Evaluation Criteria in Solid Tumors (RECIST), version 1.1. AR was defined as disease progression after PR or CR to pembrolizumab therapy based on RECIST, version 1.1.

### Oligoprogression, number of organs with progressive lesions, 2nd PFS, and post-progression survival (PPS)

Oligoprogression is a clinical state where tumor progression occurs in one or limited number of metastatic sites following previous systemic therapy wherein an initial response was observed. Previous studies have indicated that the concept of oligoprogression should be differentiated from oligometastases [12–14]. The concept of oligoprogression has been mainly proposed for patients with NSCLC with driver oncogenes and those receiving targeted therapy. The definition of oligoprogression varies among studies [15–19] and a consensus has not been reached even in ongoing clinical trials (NCT02756793 and NCT03256981). In accordance with the most popular definitions used in these studies, we defined oligoprogression as disease progression in up to 5 individual lesions. Multiple progressive lesions within a single organ or multiple progressive lymph nodes even within a single station of mediastinum were counted separately during radiological identification. Progression in truly unmeasurable lesions (such as pleural effusion, pericardiac effusion, leptomeningeal disease, etc.) was considered as progression in infinite numbers of lesions. In contrast, for determining the number of organs with progressive lesions, multiple progressive lesions within a single organ were compiled as progression in one organ. Moreover, thoracic, neck, or abdominal lymph nodes were considered as separate organs for each region in accordance with a previous study [11].

The 2nd PFS was defined as the time period between the 1st progressive disease (PD) and the 2nd PD (as defined by RECIST, considering the lesions at 1st PD as baseline) for patients who continued pembrolizumab treatment after the 1st PD. PPS was defined as the time period between the 1st PD and death of the patient regardless of receiving pembrolizumab after 1st PD.

### Statistical analyses

We described continuous variables as mean and standard deviation (SD) and categorical variables as number and percent. PFS, 2nd PFS, OS, and PPS were calculated with Kaplan–Meier estimates, and compared using the log-rank test. We defined shorter durable responses as shorter PFS despite BOR of PR/CR. To investigate the potential factors associated with shorter durable responses, we constructed univariate Cox proportional hazard models for all the clinically relevant factors (age, sex, smoking status, ECOG PS, stage, the number of organs with metastatic lesions, the presence of specific metastatic organs [pleural effusion, bone, brain, adrenal grand and liver], and early immune-related adverse events [irAEs]) as identified by previous studies on ICIs [4, 6–9, 20–23]. For the number of metastatic organs, the cutoff was set to ≥3 or < 3, as described in previous studies [20, 21]. In accordance with our previous study, we defined early irAEs as AEs with a potential immune-mediated etiology that may require immune-modulating or endocrine therapy (such as rash, pyrexia, interstitial lung disease, hypothyroidism, etc.) occurring within 3 weeks after commencement of pembrolizumab [24]. Because of small number of events, we did not perform the multivariate models. For analyses, a two-tailed *P* value of < .05 was considered significant. Statistical analyses were conducted using JMP software (version 14; SAS Institute, Cary, NC, USA).

## Results

### Treatment outcomes of study patients.

The clinical characteristics and treatment outcomes of 174 patients with NSCLC with PD-L1 TPS of ≥50% who received first-line pembrolizumab between February 1, 2017 and April 31, 2018 are summarized in eTable 1 and eFigure 1 in the Supplement, respectively. Among these, a total of 88 patients responding to first-line pembrolizumab therapy were included in the present study (Table 1).

**Table 1:** Patient characteristics

Characteristics	(***n*** = 88)
Age (years, mean ± SD)	69.4 ± 8.8
Sex, n (%)	
male	74 (84)
female	14 (16)
Smoking status, n (%)	
never smoker	10 (11)
smoker (current or former)	78 (89)
ECOG PS, n (%)	
0–1	75 (85)
2–4	13 (15)
Histology, n (%)	
Squamous	24 (27)
Non-squamous	64 (73)
Stage, n (%)	
III B	21 (24)
IV	67 (76)
*EGFR,* n (%)	
mutant	3 (3)
wild type	78 (89)
not investigated	7 (8)
*ALK,* n (%)	
rearranged	0
not rearranged	80 (91)
not investigated	8 (9)
Number of metastatic organs, n (%)	
< 3	72 (82)
≥ 3	16 (18)
Metastatic organs, n (%)	
Pleural effusion or dissemination	21 (24)
Bone	26 (30)
Brain	11 (13)
Adrenal grand	14 (16)
Liver	14 (16)

*ALK* anaplastic lymphoma kinase, *ECOG PS* Eastern Cooperative Oncology Group performance status, *EGFR* epidermal growth factor receptor, *SD* standard deviation

**Fig. 1:**
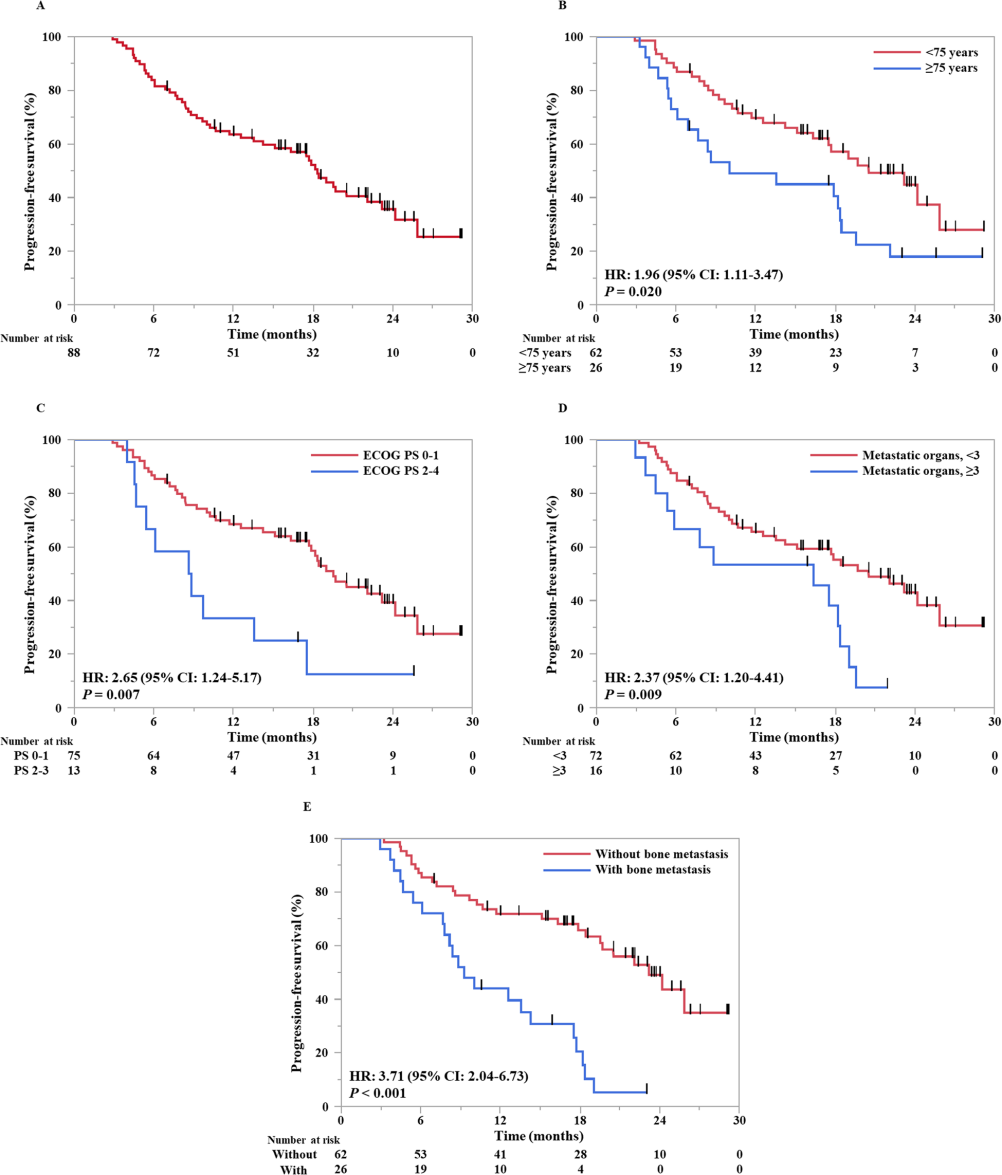
Progression-free survival in patients with non-small cell lung cancer responding to pembrolizumab monotherapy. All patients (**a**), patients stratified by age (**b**), ECOG PS (**c**), and number of metastatic organs (**d**), presence of bone metastasis (**e**). HR, hazard ratio; CI, confidence interval; PS, performance status; ECOG PS, Eastern Cooperative Oncology Group performance status. Generated using JMP software (version 14; SAS Institute, Cary, NC, USA)

During the median follow-up of 19.8 months (range: 5.4–29.2) for all the 88 patients, 46 (52%) developed AR. The patient response is summarized as a flow chart in eFigure 2 in the Supplement. The median PFS of the study patients was 18.4 months (95% CI: 13.6–22.1) (Fig. 1a). The median duration of follow-up for the patients with and without AR was 18.9 months (range: 5.4–28.5) and 22.0 months (range: 10.0–29.2), respectively. The OS data was immature, because only 21 events (24%) had occurred by the date of data cutoff.

### Clinical factors associated with shorter durable responses of pembrolizumab

The following groups of patients were significantly associated with shorter PFS: old age (≥75 years; median, 10.0 versus 20.6 months; hazard ratio [HR], 1.96; 95% CI, 1.11–3.47; *P* = .020), poor ECOG PS (2–4; median, 8.7 versus 19.6 months; HR, 2.65; 95% CI, 1.24–5.17; *P* = .007), with at least 3 metastatic organs (median, 16.4 versus 20.6 months; HR, 2.37; 95% CI, 1.20–4.41; *P* = .009), and with bone metastasis (median, 9.3 versus 23.2 months; HR, 3.71, 95% CI, 2.04–6.73; *P* < .001) (Table 2). The Kaplan–Meier curves are shown in Fig. 1b-e. Other clinical factors, such as sex, smoking status, histology, stage, metastatic organs other than bone, and early irAEs did not show any significant association with PFS (eFigure 3 in the Supplement).

**Table 2:** Univariate analyses for progression-free survival

Characteristics	No. (%)	Median PFS, month	HR (95% CI)	***P*** value
	(***n*** = 88)			
Age, years				
≥ 75	26 (30)	10.0	1.96 (1.11–3.47)	0.020*
< 75	62 (70)	20.6	Reference	
Sex				
female	14 (16)	17.5	1.02 (0.42–2.14)	0.955
male	74 (84)	18.4	Reference	
Smoking status				
smoker (current or former)	78 (89)	18.2	1.25 (0.57–3.29)	0.615
never smoker	10 (11)	18.4	Reference	
ECOG PS				
2–4	13 (15)	8.7	2.65 (1.24–5.17)	0.007*
0–1	75 (85)	19.6	Reference	
Histology				
Squamous	24 (27)	15.2	1.17 (0.63–2.17)	0.624
Non-squamous	64 (73)	18.1	Reference	
Stage				
IV	67 (76)	17.7	1.19 (0.63–2.45)	0.605
IIIB	21 (24)	19.7	Reference	
Pleural effusion or dissemination				
present	21 (24)	18.5	0.88 (0.47–1.63)	0.685
absent	67 (76)	18.2	Reference	
Bone metastasis				
present	26 (30)	9.3	3.71 (2.04–6.73)	< 0.001*
absent	62 (70)	23.2	Reference	
Brain metastasis				
present	11 (13)	19.6	0.93 (0.39–2.18)	0.858
absent	77 (88)	18.4	Reference	
Adrenal grand metastasis				
present	14 (16)	18.4	0.95 (0.44–2.02)	0.886
absent	74 (84)	18.2	Reference	
Liver metastasis				
present	14 (16)	19.6	0.78 (0.35–1.75)	0.543
absent	74 (84)	17.9	Reference	
Number of metastatic organs				
≥ 3	16 (18)	16.4	2.37 (1.20–4.41)	0.009*
< 3	72 (82)	20.6	Reference	
Early irAEs				
present	41 (47)	17.5	1.05 (0.60–1.84)	0.857
absent	47 (53)	19.6	Reference	

* *P* < 0.05

*PFS* progression-free survival, *ECOG PS* Eastern Cooperative Oncology Group performance status, *irAE* immune-related adverse event, *HR* hazard ratio, *PFS* progression-free survival

### Patterns of AR

**Fig. 2:**
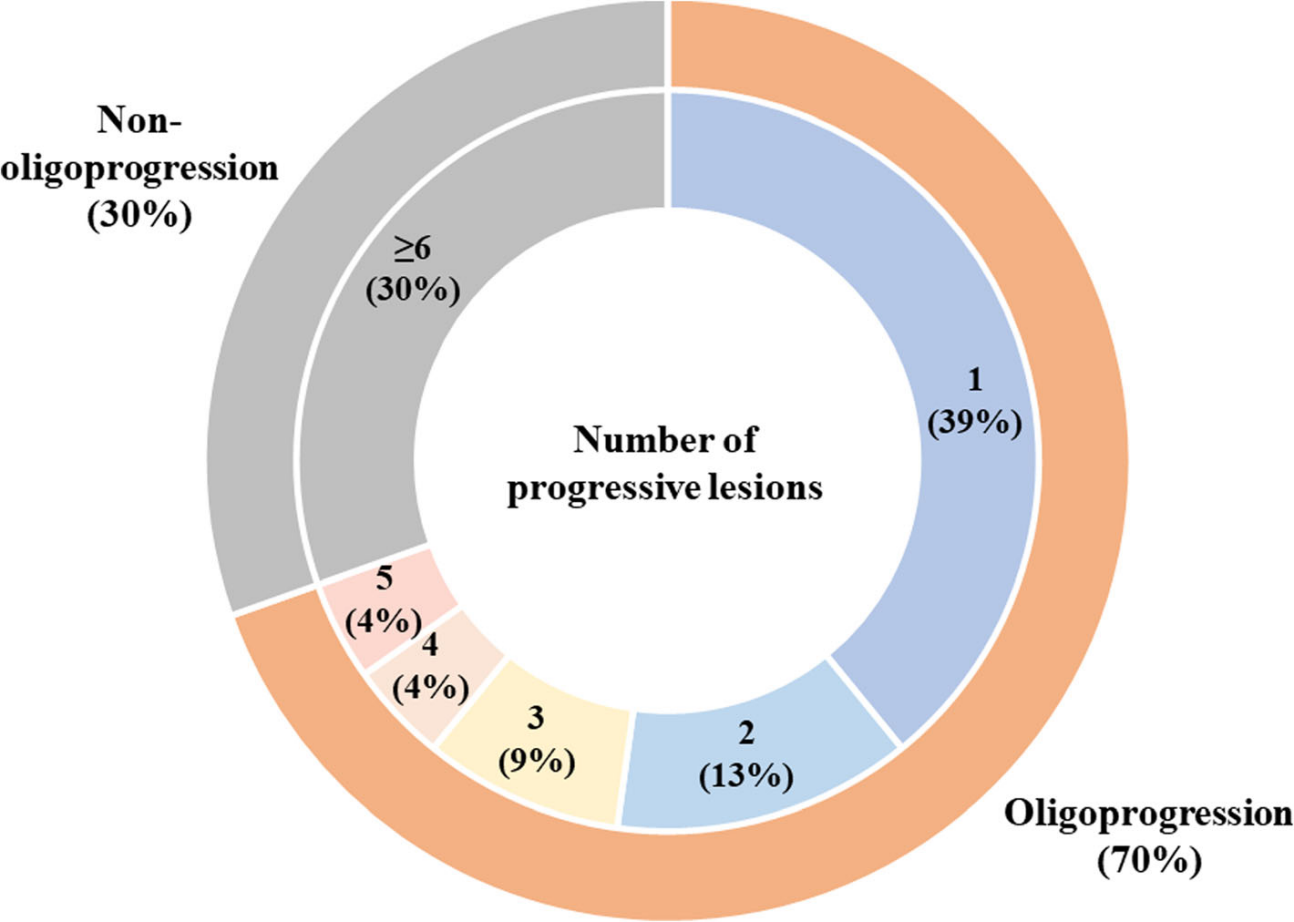
Pie-chart summarizing the number of progressive lesions in patients with acquired resistance

The numbers of individual progressive lesions are summarized in Fig. 2. Among 46 patients with AR, 18 (39%) patients had one progressive lesion, 6 (13%) had 2 lesions, 4 (9%) had 3 lesions, 2 (4%) had 4 lesions, 2 (4%) had 5 lesions, and 14 (30%) had at least 6 progressive lesions. In total, oligoprogression was seen in 32 (70%) patients.

Patients with AR were classified into three categories: progression in only pre-existing (before commencing pembrolizumab) lesions, progression in only new lesions, or both. In oligoprogressive AR patients 21/7/4 patients showed the above progression patterns and in non-oligoprogressive AR patients 6/3/5 patients showed as such.

Regarding the number of organs with progressive lesions, 26 (57%) of 46 patients developed it in one organ, 11 (24%) in two organs, and 9 (20%) in three or more organs (eFigure 4 in the Supplement). The common organs associated with AR were the lungs (*n* = 22, 52%), thoracic lymph node (*n* = 16, 35%), and bone (*n* = 10, 22%). A total of 20 patients (43%) developed AR in the lymph nodes.

### Treatment and prognosis of patients with AR

Among the 46 patients with AR, 19 (41%) received subsequent platinum-based doublet (with or without anti-vascular endothelial growth factor agent) therapy after pembrolizumab, 7 (15%) continued pembrolizumab therapy beyond 1st PD, 6 (13%) received subsequent monotherapy of cytotoxic agents, 1 (2%) received EGFR-tyrosine kinase inhibitor therapy, and 13 (28%) received only best-supportive care. In addition, 7 patients (15%), who developed AR as oligoprogression, received local ablative therapy (radiation therapy) for all lesions of AR. Four patients continued pembrolizumab and did not receive local ablative therapy, and none of them showed tumor shrinkage again and that PD was considered as true PD (not pseudoprogression).

**Fig. 3:**
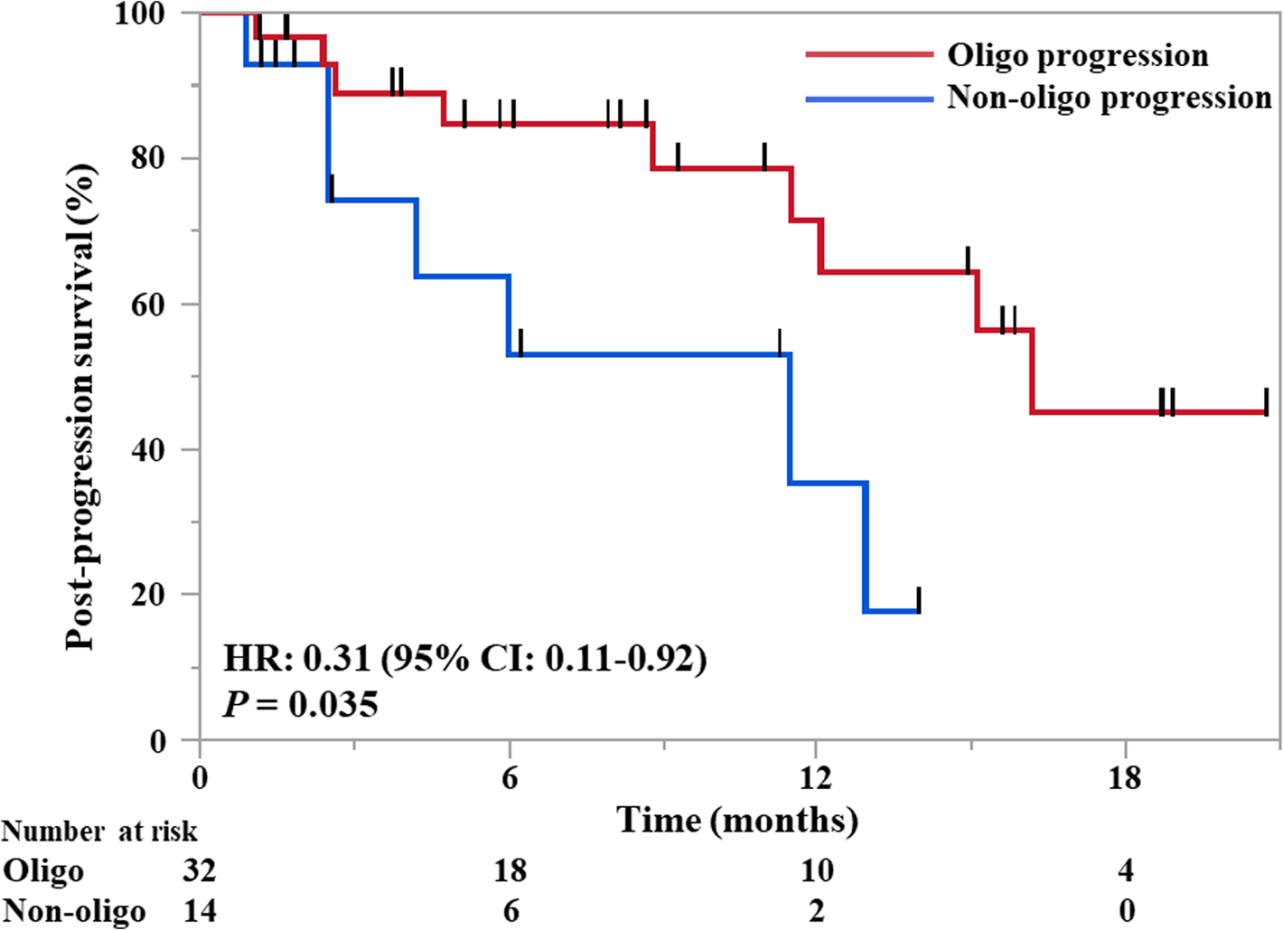
Post-progression survival of patients with acquired resistance, stratified based on presence or absence of oligoprogression. HR, hazard ratio; CI, confidence interval. Generated using JMP software (version 14; SAS Institute, Cary, NC, USA)

The PPS in all the patients with AR (*n* = 46) was 15.1 months (95% CI: 11.5-not reached). It was significantly longer in patients with oligoprogressive AR than in those with non-oligoprogressive AR: 16.2 months, (95% CI: 11.5-not reached) versus 11.5 months (95% CI: 2.5-not reached), HR, 0.31; 95% CI, 0.11–0.92; *P* = .035 (Fig. 3). Moreover, the median 2nd PFS was not reached (95% CI: 7.7-not reached) in patients with AR who received local ablative radiation therapy for all lesions of AR and continued pembrolizumab therapy beyond 1st progression (*n =* 4) (eFigure 5 in the Supplement).

## Discussion

We revealed that factors such as old age, poor PS, or metastatic organs ≥3 were associated with shorter durable responses of first-line pembrolizumab monotherapy in NSCLC patients who showed response to the therapy. Further, oligoprogression was found to be relatively common and associated with better prognosis. To the best of our knowledge, this is the first study to explore the clinical factors associated with the shorter durable responses of ICI therapy and the largest cohort study performed in patients with AR.

The factors that were found to be associated with shorter PFS in our study have also been reported by previous studies [20, 21, 25–28]; however, these studies mainly focused on primary resistance. To date, no study has investigated the clinical factors associated with AR to ICI therapy. Although the clinical factors associated with and mechanisms underlying AR to ICI therapy are not fully understood, old age and poor PS (frailty) have been shown to be associated with immunosuppressive activity, which inhibits ICI-induced activation of the immune system [29, 30]. In addition, a high antigen burden is shown to have negative effect on the activation of T-cells, as indicated by a previous study on viral infection [31].

We revealed that the majority of AR occurred as oligoprogression (70%). In previous studies, the proportion of oligoprogression was reported to be 15–47% in patients with NSCLC with driver oncogenes and who showed AR to targeted therapy [13, 17, 18], which is lower than that reported in our study. This may be attributed to the difference in the definition of oligoprogression and in the treatment class. The definition of oligoprogression has yet to reach consensus, and we adopted one definition used in multiple studies, as mentioned above. Stratifying by this definition, patients with oligoprogressive AR showed longer PPS, indicative of the validity of our definition in discriminating distinct populations. Further discussions on the definition are warranted to plan clinical trials for oligoprogressive AR patients and develop better treatment strategies for these patients.

Oligoprogression after ICI therapy has been reported previously only by an individual report that considered a smaller number of samples (*n* = 26) [11]. However, our study included a greater number of samples and identified patients with clearly defined oligoprogression. Moreover, the previous study included patients regardless of therapeutic agents (including PD-L1 and cytotoxic T-lymphocyte-associated antigen 4 [CTLA-4] axis inhibitor alone or in combination) and treatment lines [11]. In contrast, our study included more homogenous patients receiving only first-line pembrolizumab monotherapy.

The current study further revealed that many patients developed AR in only one organ (57%), which is in agreement with an earlier study showing development of AR in only one organ for 54% of the patients [11]. In addition, the previous report emphasized the development of AR in the lymph nodes for majority of the patients (77%). However, in the current study, only 43% of the patients developed AR in the lymph nodes. A probable reason for this discrepancy could be attributed to the difference in treatment lines and agents. The patterns of AR to ICI therapy need to be evaluated in future studies.

The survival analysis of patients with AR revealed that oligoprogressive patients showed longer PPS than their non-oligo (or systemic) progressive counterparts. Moreover, although limited in number, oligoprogressive patients who received local ablative radiotherapy and continued pembrolizumab beyond progression showed promising 2nd PFS in our cohort. Similarly, local ablative therapy after targeted therapy for oligoprogressive NSCLC patients has shown clinical efficacy previously [17, 19]. Local ablative radiation therapy in combination with ICIs has been shown to be more promising owing to synergetic effect called abscopal effect [32, 33], and is currently being considered in clinical trials [34, 35]. Because of the high frequency of oligoprogressive disease, further studies are needed to investigate the efficacy of local ablative therapy in patients with oligoprogressive AR to ICI therapy.

The mechanisms underlying AR to ICI therapy are not fully understood; however, some of them were partially in common with primary resistance and have been explained in different studies [36–39]. These mechanisms are roughly classified as follows: intrinsic cancer cell resistance, intrinsic T cell resistance, and extrinsic resistance. Intrinsic cancer cell resistance represents loss of immunogenicity of cancer cells [40], which were suggested to result from alterations, such as loss of beta-2-microglobullin (B2M) function [41, 42] and phosphatase and tensin homolog deleted on chromosome 10 (PTEN) function [43]. Intrinsic T cell resistance represents immune adaptation caused by upregulation of other immune checkpoint molecules such as CTLA-4, lymphocyte activation gene 3 (LAG-3) or T-cell immunoglobulin and mucin domain 3 (TIM-3) [44, 45]. Extrinsic resistance represents modulation of tumor microenvironment (TME) through infiltration of immunosuppressive cells, such as regulatory T cells [46] and myeloid-derived suppressor cells (MDSCs) [47]. We could not explain the reason for the high frequency and better prognosis of patients with oligoprogressive AR in our study, and the underlying mechanisms should be explored in further studies.

The treatment outcomes in our entire cohort were in agreement with those reported in previous clinical trials. The objective response rate and median PFS of first-line PD-1 axis inhibitor monotherapy in patients with NSCLC with PD-L1 TPS of ≥50% were reported to be 37–58% and 5.6–12.5 months, respectively, in previous studies [4, 5, 48]; these values were in agreement with the findings in our entire cohort, including all the patients who received pembrolizumab. Further, the median time to response and duration of response for PD-1 axis inhibitors were reported to be 2.1–2.2 and 16.3–25.2 months, respectively, in previous phase 3 trials [5–9, 49]. These results were in agreement with our study, wherein the median PFS of patients with response to first-line pembrolizumab treatment was 18.4 months.

The present study has several limitations. First, although our study included the largest multicenter cohort of its kind and provided novel findings, it was retrospective in nature. This limitation includes the retrospective assessment of tumor responses and metastatic lesions at diagnosis of NSCLC. Second, biomarkers other than PD-L1 expression, such as tumor mutation burden, were not investigated. However, these biomarkers are currently under active investigation because they produced conflicting results regarding their clinical benefit. Third, the distribution of treatment patterns after progression was different between oligoprossive and non-oligoprogressive AR patients. The proportions of oligoprossive and non-oligoprogressive AR patients who received only the best supportive care were 22 and 43%, respectively, which may have influenced the analysis of PPS. Fourth, we focused on AR and did not collect data on primary resistance. We could not determine whether the clinical features of AR revealed were specific to AR or in common with primary resistance and AR.

## Conclusions

We revealed that old age, poor PS, or at least 3 metastatic organs were associated with shorter durable responses to pembrolizumab. Further, patients with oligoprogressive AR were relatively common and associated with better prognosis. We believe that these findings provide a scope for improving ICI therapy and suggest new directions for clinical studies.

## Supplementary Information

**Additional file 1.**

**Additional file 2 Supplementary Fig. 1.** Progression-free survival (A) and overall survival (B) in all patients who received first-line pembrolizumab. Generated using JMP software (version 14; SAS Institute, Cary, NC, USA). **Supplementary Fig. 2.** Flow chart of the study patients. **Supplementary Fig. 3.** Progression-free survival (PFS) in patients with response, stratified by sex (A), smoking history (B), histology (C), stage (D), and presence of pleural effusion or dissemination (E), brain metastasis (F), adrenal grand metastasis (G), liver metastasis (H), early immune-related adverse events (irAEs) (I). Generated using JMP software (version 14; SAS Institute, Cary, NC, USA). **Supplementary Fig. 4.** Pie-chart summarizing the organs with progressive lesions. Patients with progression in one organ (blue), 2 organs (orange), or 3 or more organs (grey). **Supplementary Fig. 5.** The 2nd progression-free survival of patients who developed acquired resistance, received local ablative therapy and pembrolizumab therapy beyond 1st PD. Generated using JMP software (version 14; SAS Institute, Cary, NC, USA).

**Additional file 3.**

## Data Availability

The datasets generated during and/or analysed during the current study are available from the corresponding author on reasonable request.

## Abbreviations

AR: Acquired resistance
B2M: Beta-2-microglobullin
BOR: Best overall response
CR: Complete response
CTLA-4: Cytotoxic T-lymphocyte-associated antigen 4
DCR: Disease control rate
ECOG PS: Eastern Cooperative Oncology Group performance status
EGFR: Epidermal growth factor receptor
ICI: Immune checkpoint inhibitor
irAE: Immune-related adverse event
LAG-3: Lymphocyte activation gene 3
MDSC: Myeloid-derived suppressor cell
NSCLC: Non-small cell lung cancer
ORR: Overall response rate
OS: Overall survival
PD-1: Programmed cell death 1
PD-L1: Programmed cell death ligand 1
PFS: Progression-free survival
PR: Partial response
PTEN: Tensin homolog deleted on chromosome 10
RECIST: Response Evaluation Criteria in Solid Tumors
TIM-3: T-cell immunoglobulin and mucin domain 3
TME: Tumor microenvironment
TPS: Tumor proportion score
