# Supplementary material for: Clinical factors associated with shorter durable response, and patterns of acquired resistance to first-line pembrolizumab monotherapy in PD-L1-positive non-small-cell lung cancer patients: a retrospective multicenter study
Source: BMC Cancer. 2021 Apr 1;21:346. doi: 10.1186/s12885-021-08048-4 (PMC8017679; doi:10.1186/s12885-021-08048-4)
Supplement: Supplementary file 1 — Additional file 1. [file 12885_2021_8048_MOESM1_ESM.docx]

| **Supplementary table 1. Patient characteristics of all patients who received first-line pembrolizumab regardless of responses** | | |
| --- | --- | --- |
| **Characteristics** | | **(n=174)** |
| age (years, mean ± SD) | | 69.3 ± 9.6 |
| Sex, n (%) | |  |
|  | male | 143 (82) |
|  | female | 31 (18) |
| Smoking status, n (%) | |  |
|  | never smoker | 18 (10) |
|  | smoker (current or former) | 156 (90) |
| ECOG PS, n (%) | |  |
|  | 0-1 | 141 (81) |
|  | 2-4 | 33 (19) |
| Histology, n (%) | |  |
|  | Squamous | 40 (23) |
|  | Non-squamous | 134 (77) |
| Stage, n (%) | |  |
|  | III B | 31 (18) |
|  | IV | 143 (82) |
| *EGFR,* n (%) | |  |
|  | mutant | 5 (3) |
|  | wild type | 151 (87) |
|  | not investigated | 18 (10) |
| *ALK,* n (%) | |  |
|  | rearranged | 0 |
|  | not rearranged | 157 (91) |
|  | not investigated | 15 (9) |
| Metastatic organs, n (%) | |  |
|  | Pleural effusion or dissemination | 54 (32) |
|  | Bone | 51 (30) |
|  | Brain | 30 (18) |
|  | Adrenal grand | 27 (16) |
|  | Liver | 24 (14) |
| Number of metastatic organs, n (%) | |  |
|  | <3 | 137 (79) |
|  | ≥3 | 37 (21) |
| ECOG PS = Eastern Cooperative Oncology Group performance status; *EGFR* = epidermal growth factor receptor; *ALK* = anaplastic lymphoma kinase | | |
