# Supplementary material for: Clinical factors associated with shorter durable response, and patterns of acquired resistance to first-line pembrolizumab monotherapy in PD-L1-positive non-small-cell lung cancer patients: a retrospective multicenter study
Source: BMC Cancer. 2021 Apr 1;21:346. doi: 10.1186/s12885-021-08048-4 (PMC8017679; doi:10.1186/s12885-021-08048-4)
Supplement: Supplementary file 2 — Additional file 2 Supplementary Fig. 1. Progression-free survival (A) and overall survival (B) in all patients who received first-line pembrolizumab. Generated using JMP software (version 14; SAS Institute, Cary, NC, USA). Supplementary Fig. 2. Flow chart of the study patients. Supplementary Fig. 3. Progression-free survival (PFS) in patients with response, stratified by sex (A), smoking history (B), histology (C), stage (D), and presence of pleural effusion or dissemination (E), brain metastasis (F), adrenal grand metastasis (G), liver metastasis (H), early immune-related adverse events (irAEs) (I). Generated using JMP software (version 14; SAS Institute, Cary, NC, USA). Supplementary Fig. 4. Pie-chart summarizing the organs with progressive lesions. Patients with progression in one organ (blue), 2 organs (orange), or 3 or more organs (grey). Supplementary Fig. 5. The 2nd progression-free survival of patients who developed acquired resistance, received local ablative therapy and pembrolizumab therapy beyond 1st PD. Generated using JMP software (version 14; SAS Institute, Cary, NC, USA). [file 12885_2021_8048_MOESM2_ESM.pptx]

## Slide 1
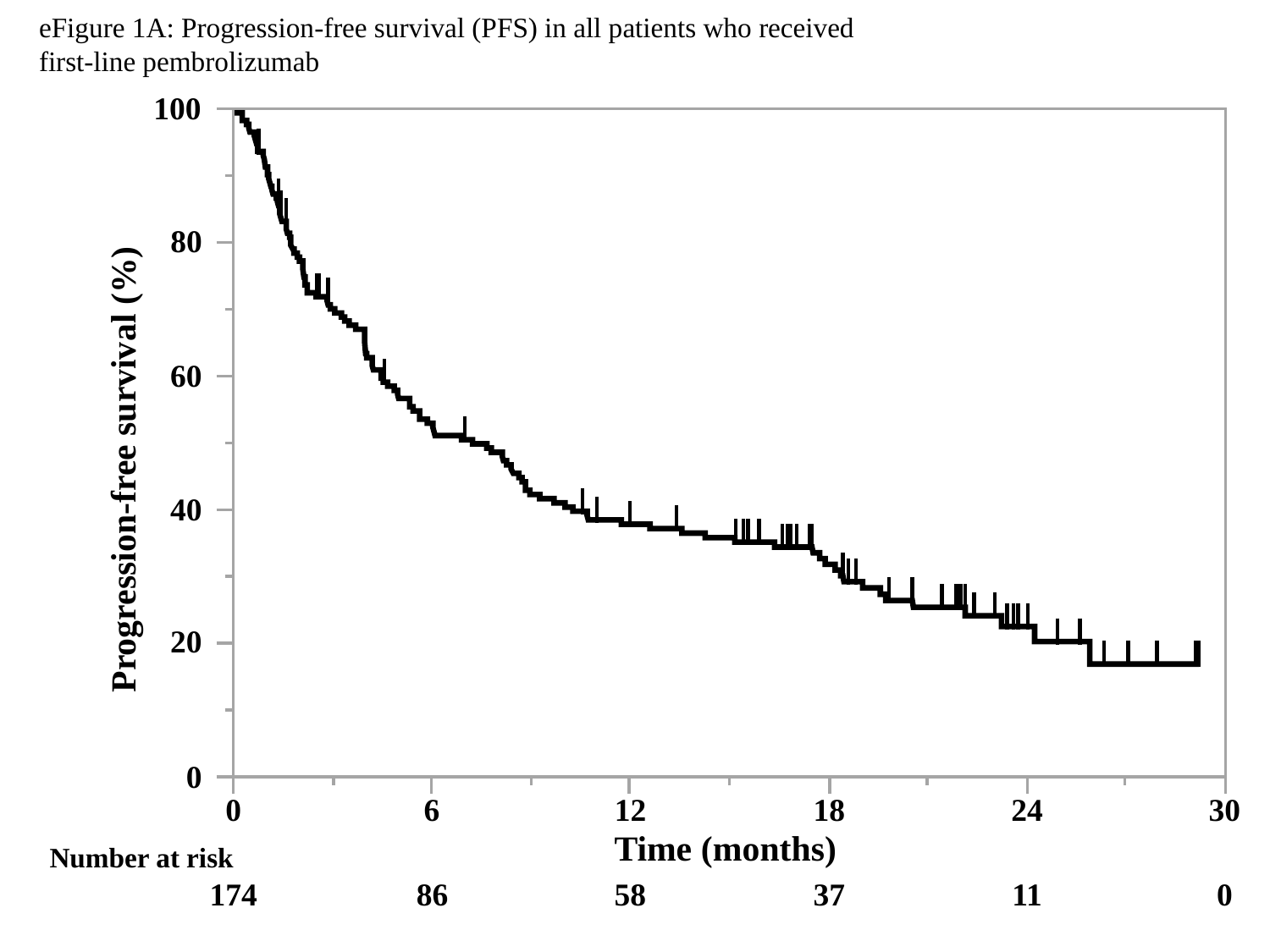

eFigure 1A: Progression-free survival (PFS) in all patients who received
first-line pembrolizumab
100
Progression-free survival (%)
80
60
40
20
0
0
6
12
18
24
30
Time (months)
Number at risk
174
86
58
37
11
0

## Slide 2
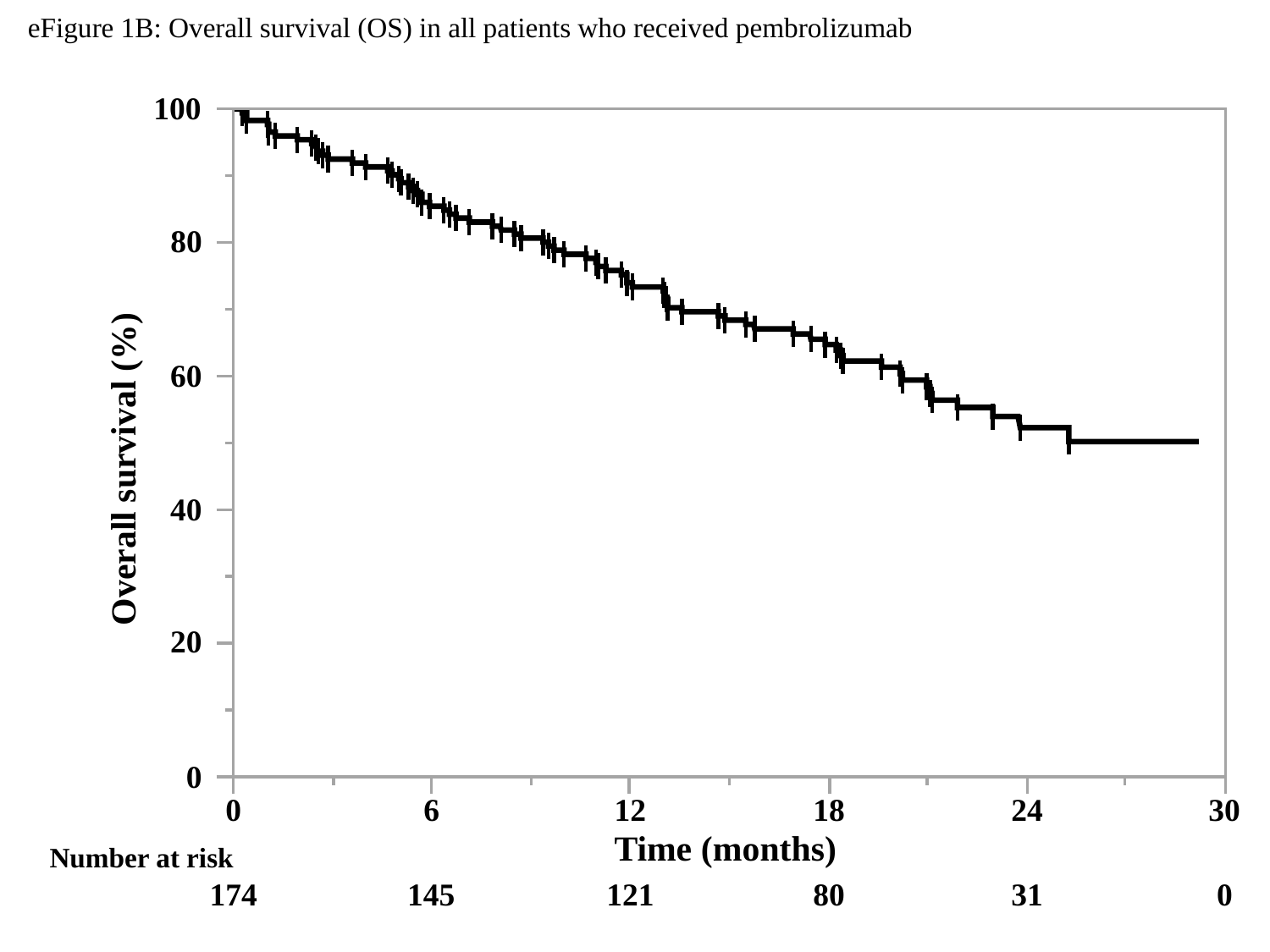

eFigure 1B: Overall survival (OS) in all patients who received pembrolizumab
100
Overall survival (%)
80
60
40
20
0
0
6
12
18
24
30
Time (months)
Number at risk
174
145
121
80
31
0

## Slide 3
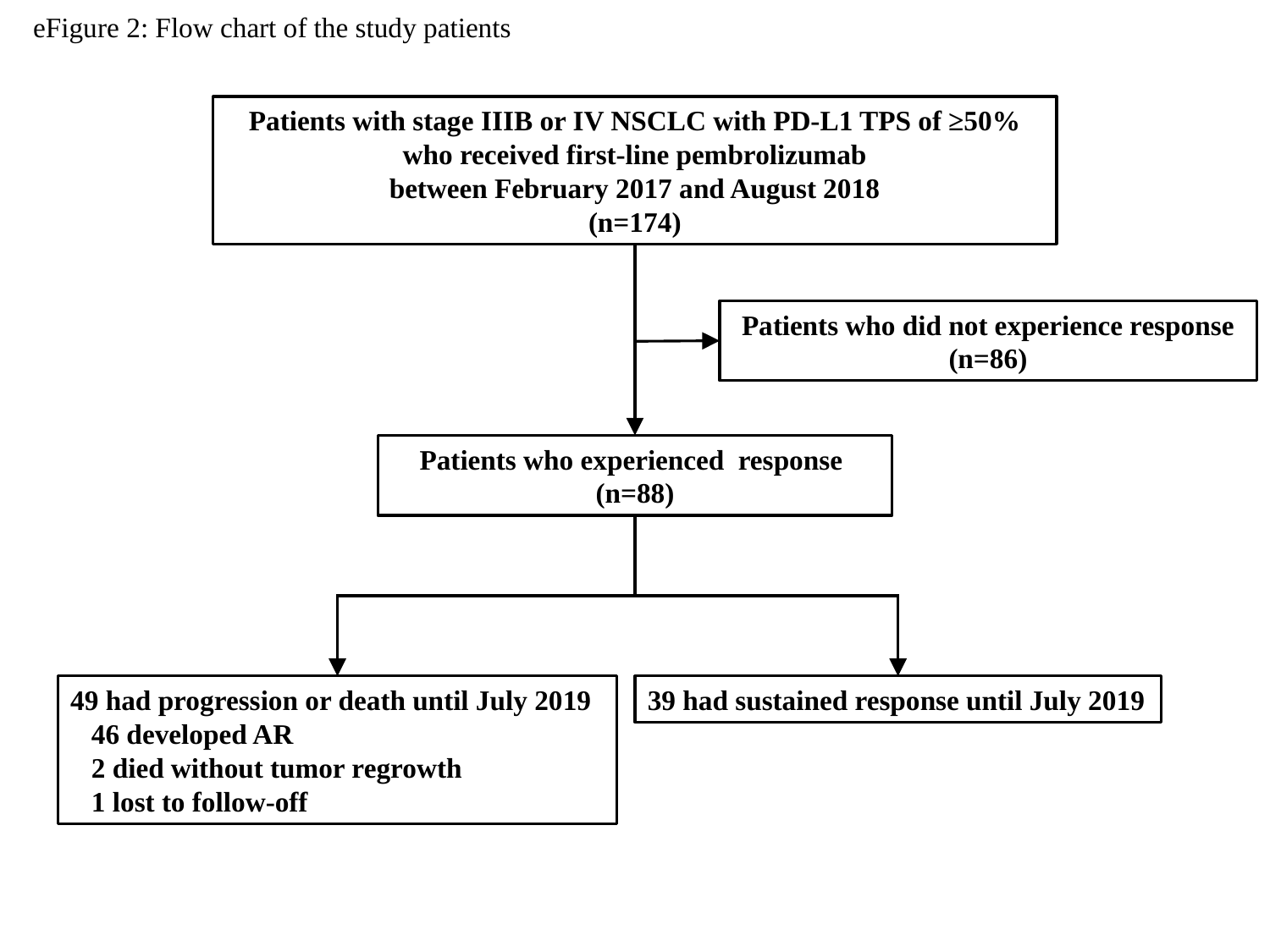

eFigure 2: Flow chart of the study patients
Patients with stage IIIB or IV NSCLC with PD-L1 TPS of ≥50%
who received first-line pembrolizumab
between February 2017 and August 2018
(n=174)
Patients who did not experience response (n=86)
Patients who experienced response
(n=88)
49 had progression or death until July 2019
 46 developed AR
 2 died without tumor regrowth
 1 lost to follow-off
39 had sustained response until July 2019

## Slide 4
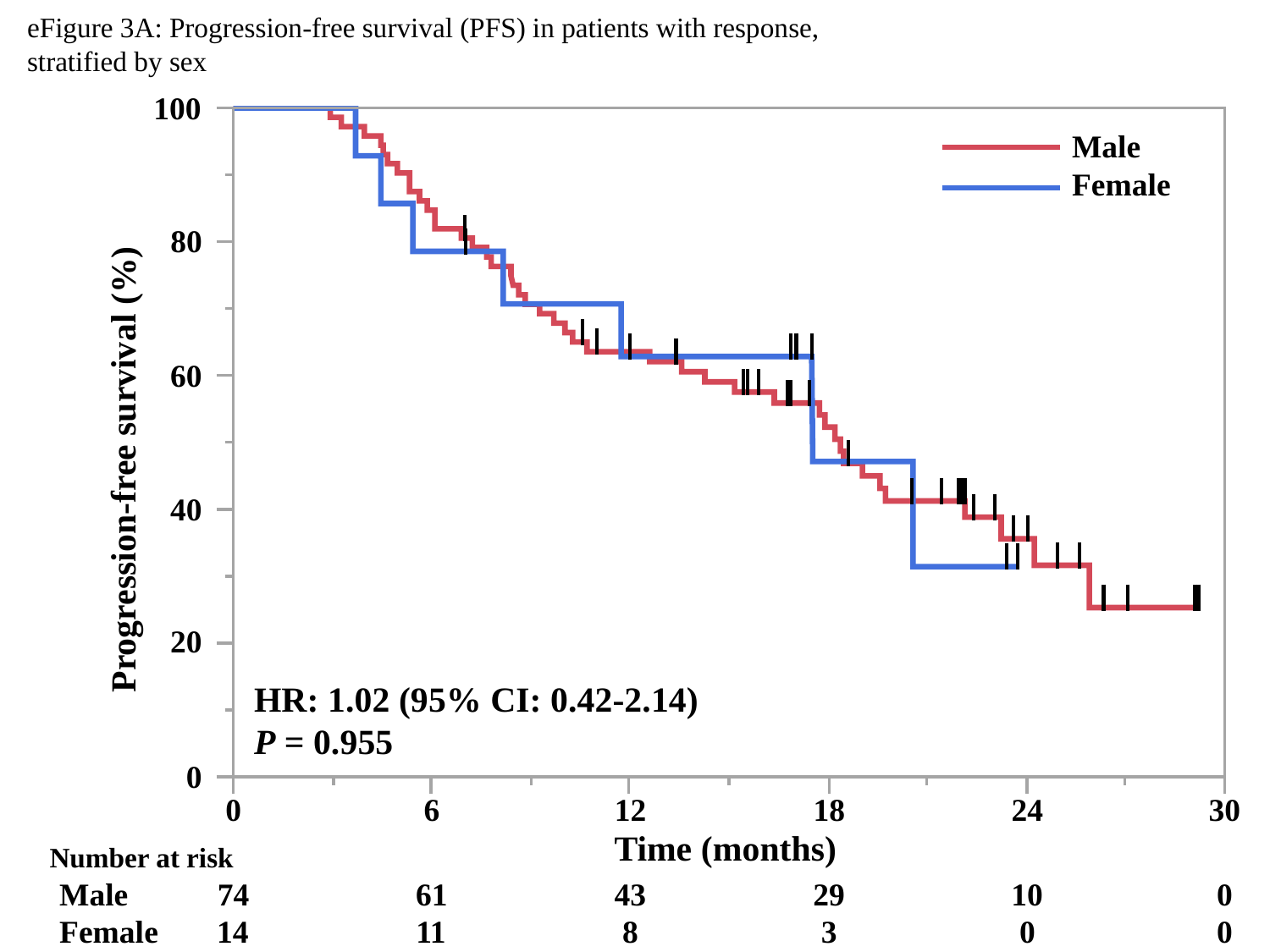

eFigure 3A: Progression-free survival (PFS) in patients with response,
stratified by sex
100
Male
Female
Progression-free survival (%)
80
60
40
20
HR: 1.02 (95% CI: 0.42-2.14)
P = 0.955
0
0
6
12
18
24
30
Time (months)
Number at risk
Male
74
61
43
29
10
0
Female
14
11
8
3
0
0

## Slide 5
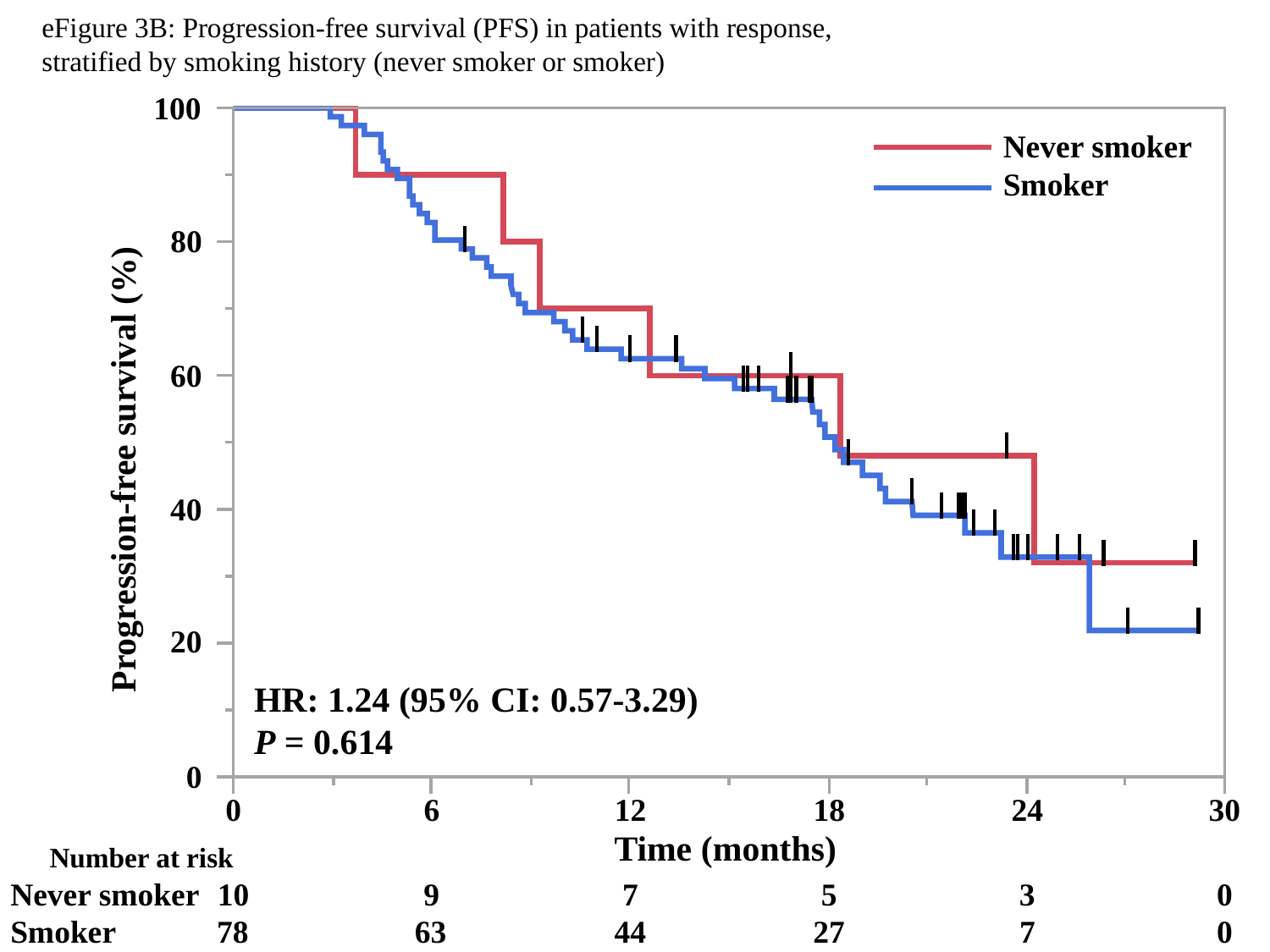

eFigure 3B: Progression-free survival (PFS) in patients with response,
stratified by smoking history (never smoker or smoker)
100
Never smoker
Smoker
Progression-free survival (%)
80
60
40
20
HR: 1.24 (95% CI: 0.57-3.29)
P = 0.614
0
0
6
12
18
24
30
Time (months)
Number at risk
Never smoker
10
9
7
5
3
0
Smoker
78
63
44
27
7
0

## Slide 6
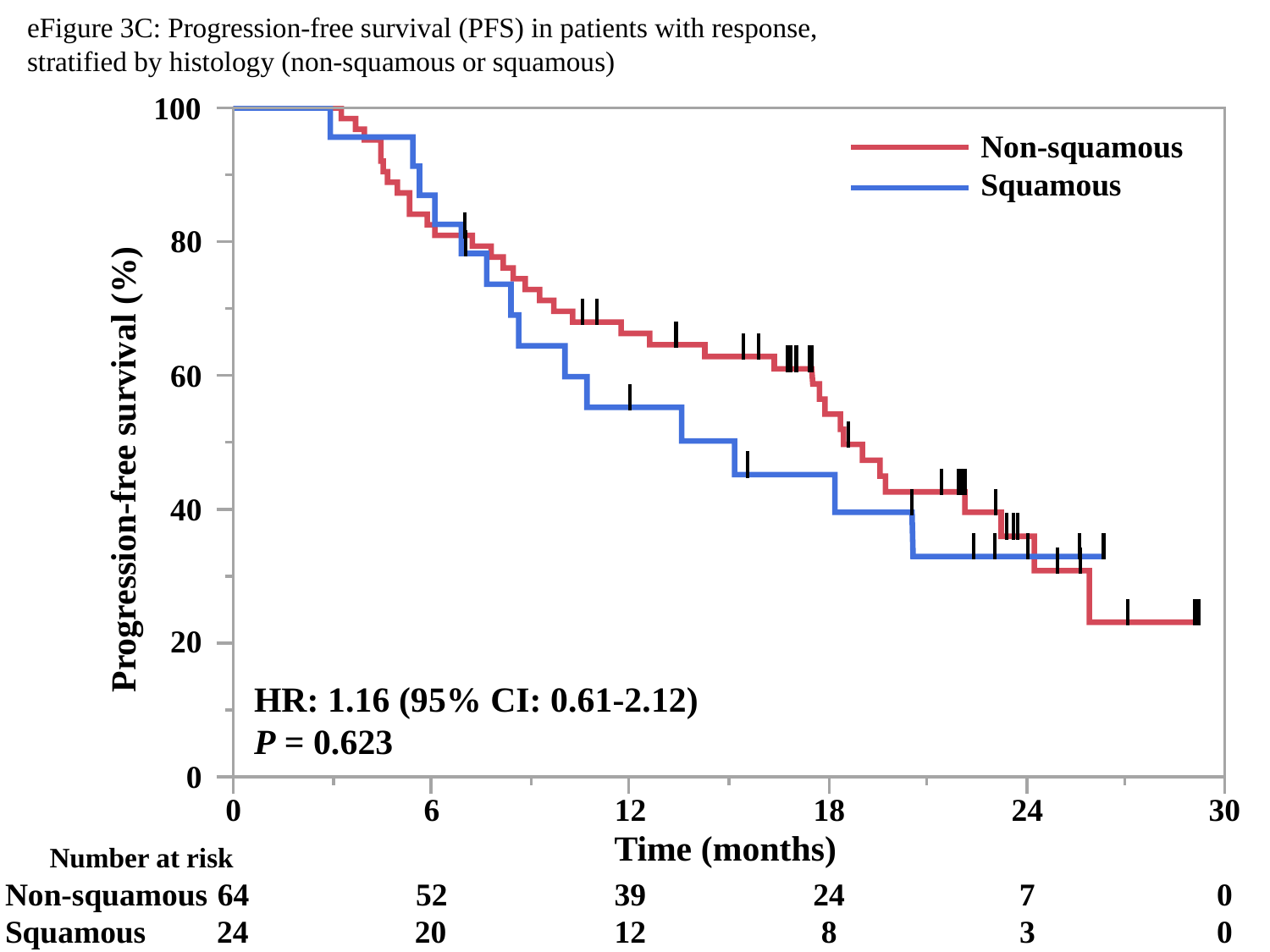

eFigure 3C: Progression-free survival (PFS) in patients with response,
stratified by histology (non-squamous or squamous)
100
Non-squamous
Squamous
Progression-free survival (%)
80
60
40
20
HR: 1.16 (95% CI: 0.61-2.12)
P = 0.623
0
0
6
12
18
24
30
Time (months)
Number at risk
Non-squamous
64
52
39
24
7
0
Squamous
24
20
12
8
3
0

## Slide 7
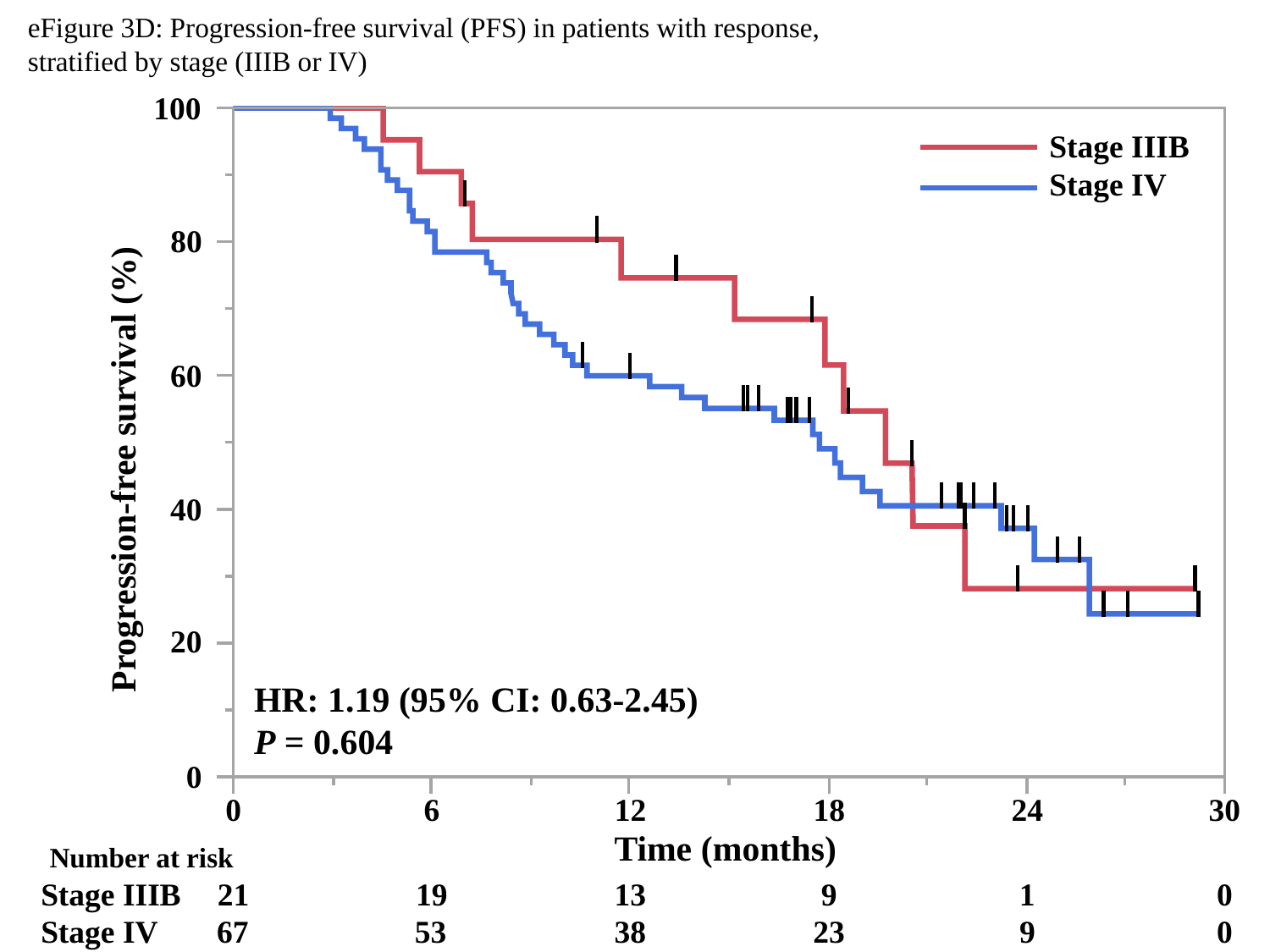

eFigure 3D: Progression-free survival (PFS) in patients with response,
stratified by stage (IIIB or IV)
100
Stage IIIB
Stage IV
Progression-free survival (%)
80
60
40
20
HR: 1.19 (95% CI: 0.63-2.45)
P = 0.604
0
0
6
12
18
24
30
Time (months)
Number at risk
Stage IIIB
21
19
13
9
1
0
Stage IV
67
53
38
23
9
0

## Slide 8
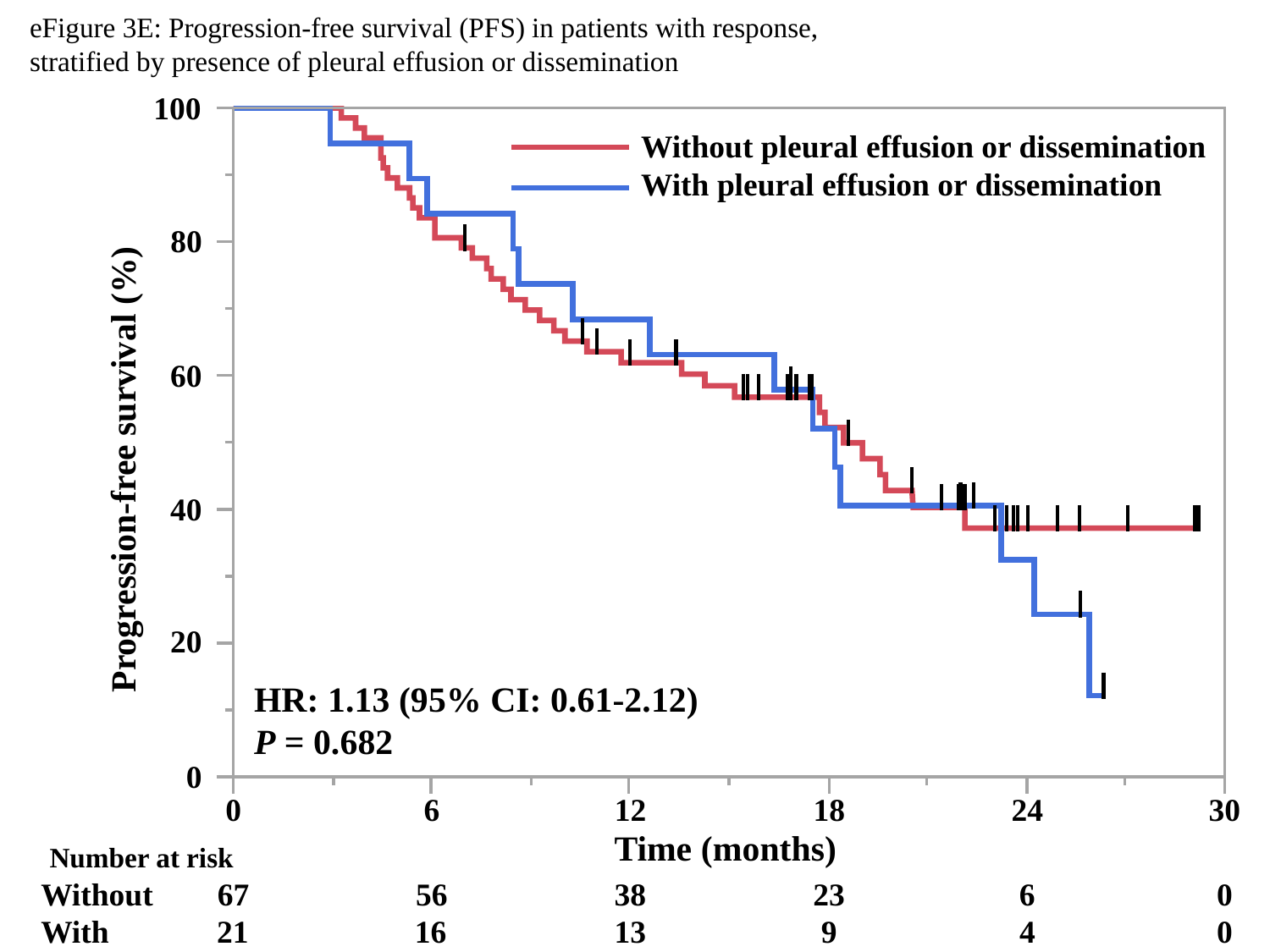

eFigure 3E: Progression-free survival (PFS) in patients with response,
stratified by presence of pleural effusion or dissemination
100
Without pleural effusion or dissemination
With pleural effusion or dissemination
Progression-free survival (%)
80
60
40
20
HR: 1.13 (95% CI: 0.61-2.12)
P = 0.682
0
0
6
12
18
24
30
Time (months)
Number at risk
Without
67
56
38
23
6
0
With
21
16
13
9
4
0

## Slide 9
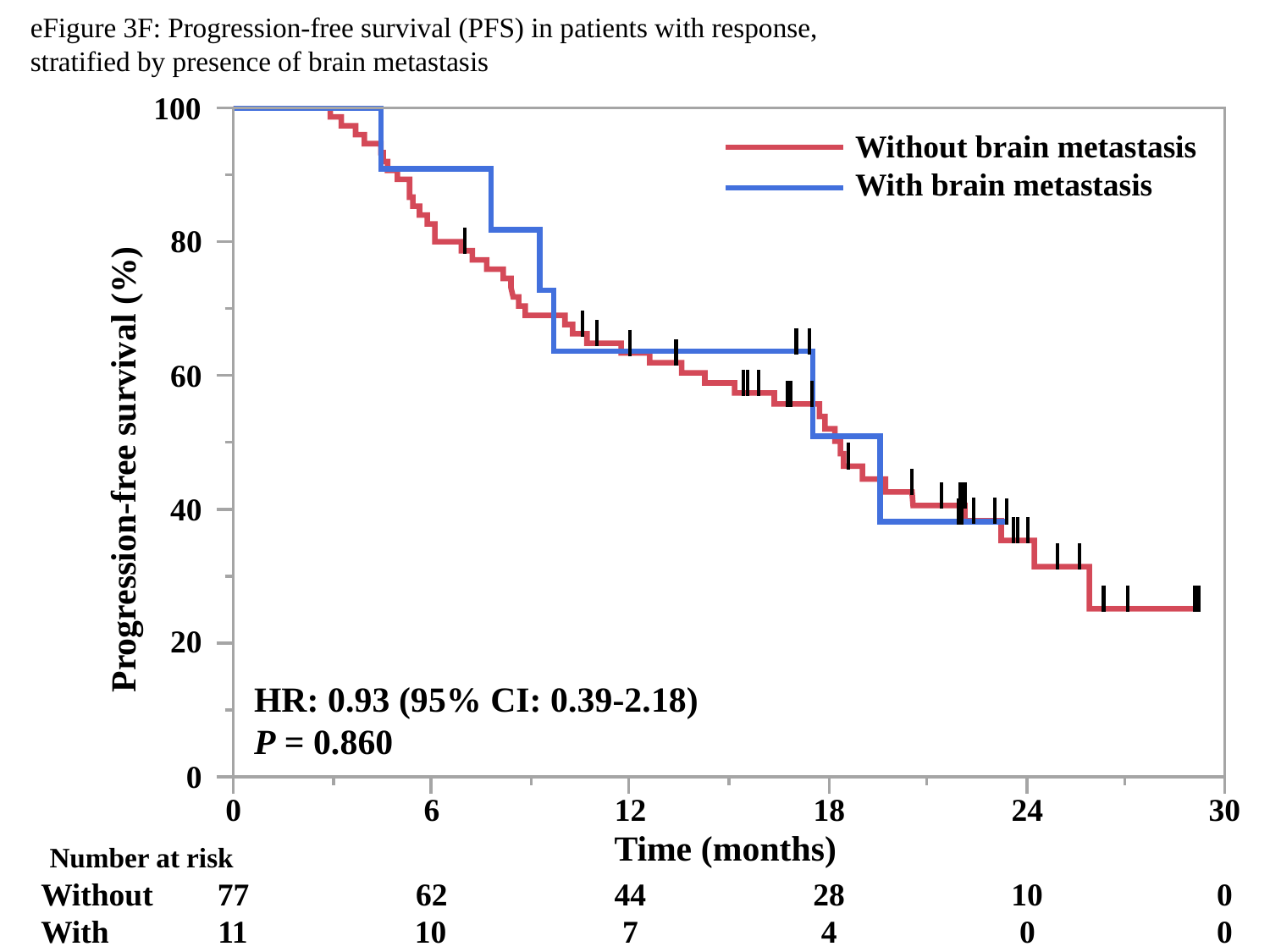

eFigure 3F: Progression-free survival (PFS) in patients with response,
stratified by presence of brain metastasis
100
Without brain metastasis
With brain metastasis
Progression-free survival (%)
80
60
40
20
HR: 0.93 (95% CI: 0.39-2.18)
P = 0.860
0
0
6
12
18
24
30
Time (months)
Number at risk
Without
77
62
44
28
10
0
With
11
10
7
4
0
0

## Slide 10
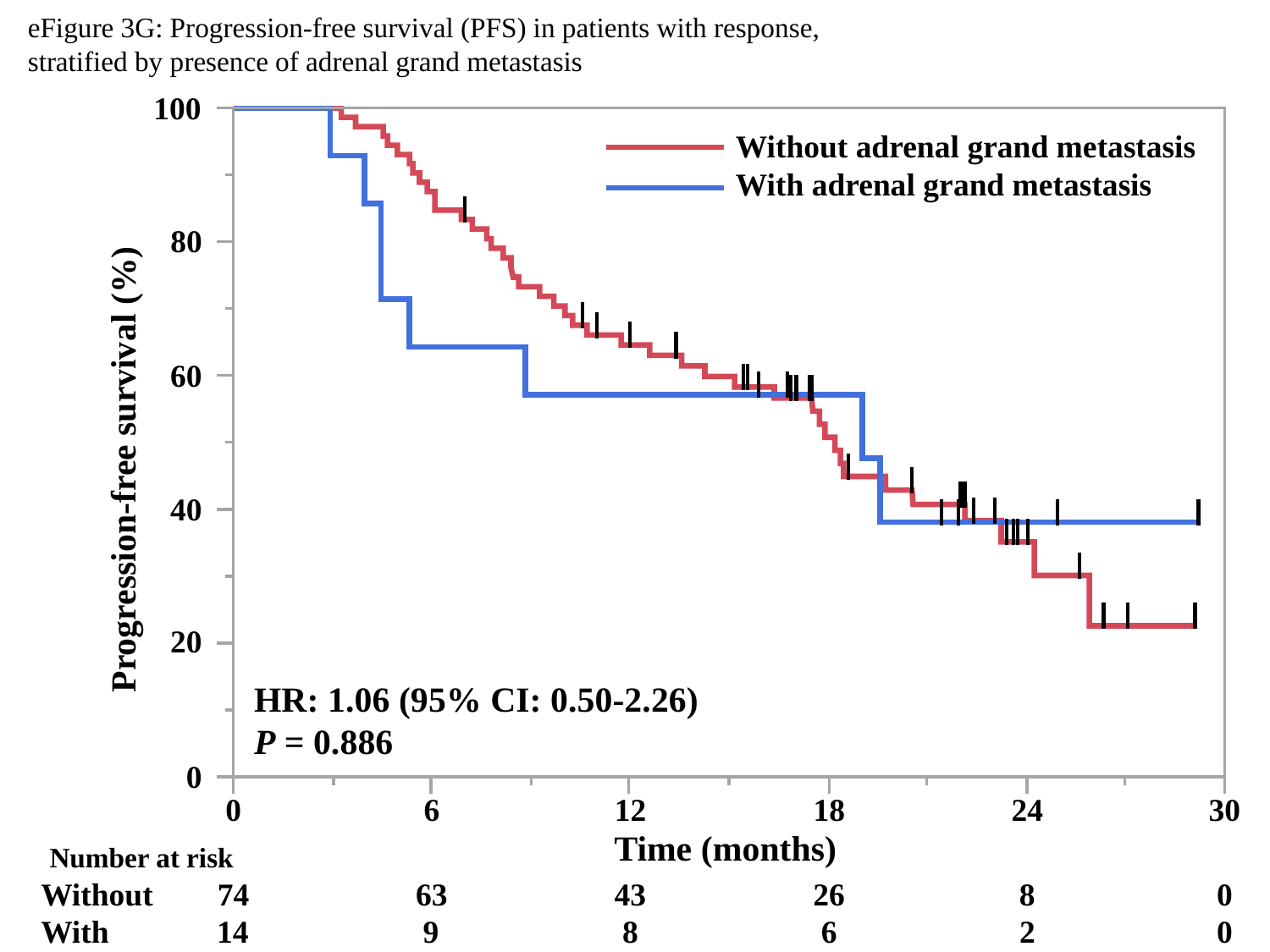

eFigure 3G: Progression-free survival (PFS) in patients with response,
stratified by presence of adrenal grand metastasis
100
Without adrenal grand metastasis
With adrenal grand metastasis
Progression-free survival (%)
80
60
40
20
HR: 1.06 (95% CI: 0.50-2.26)
P = 0.886
0
0
6
12
18
24
30
Time (months)
Number at risk
Without
74
63
43
26
8
0
With
14
9
8
6
2
0

## Slide 11
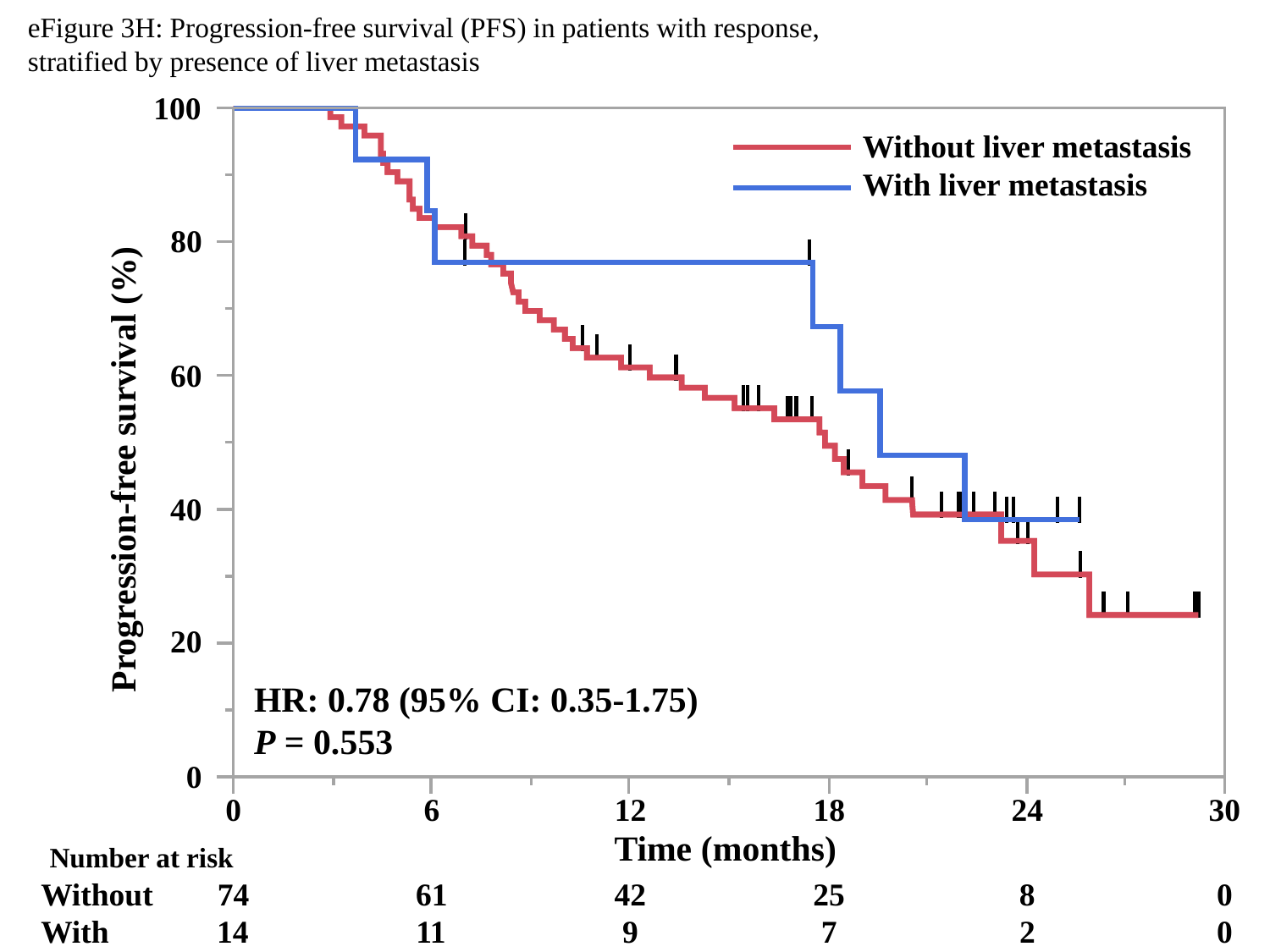

eFigure 3H: Progression-free survival (PFS) in patients with response,
stratified by presence of liver metastasis
100
Without liver metastasis
With liver metastasis
Progression-free survival (%)
80
60
40
20
HR: 0.78 (95% CI: 0.35-1.75)
P = 0.553
0
0
6
12
18
24
30
Time (months)
Number at risk
Without
74
61
42
25
8
0
With
14
11
9
7
2
0

## Slide 12
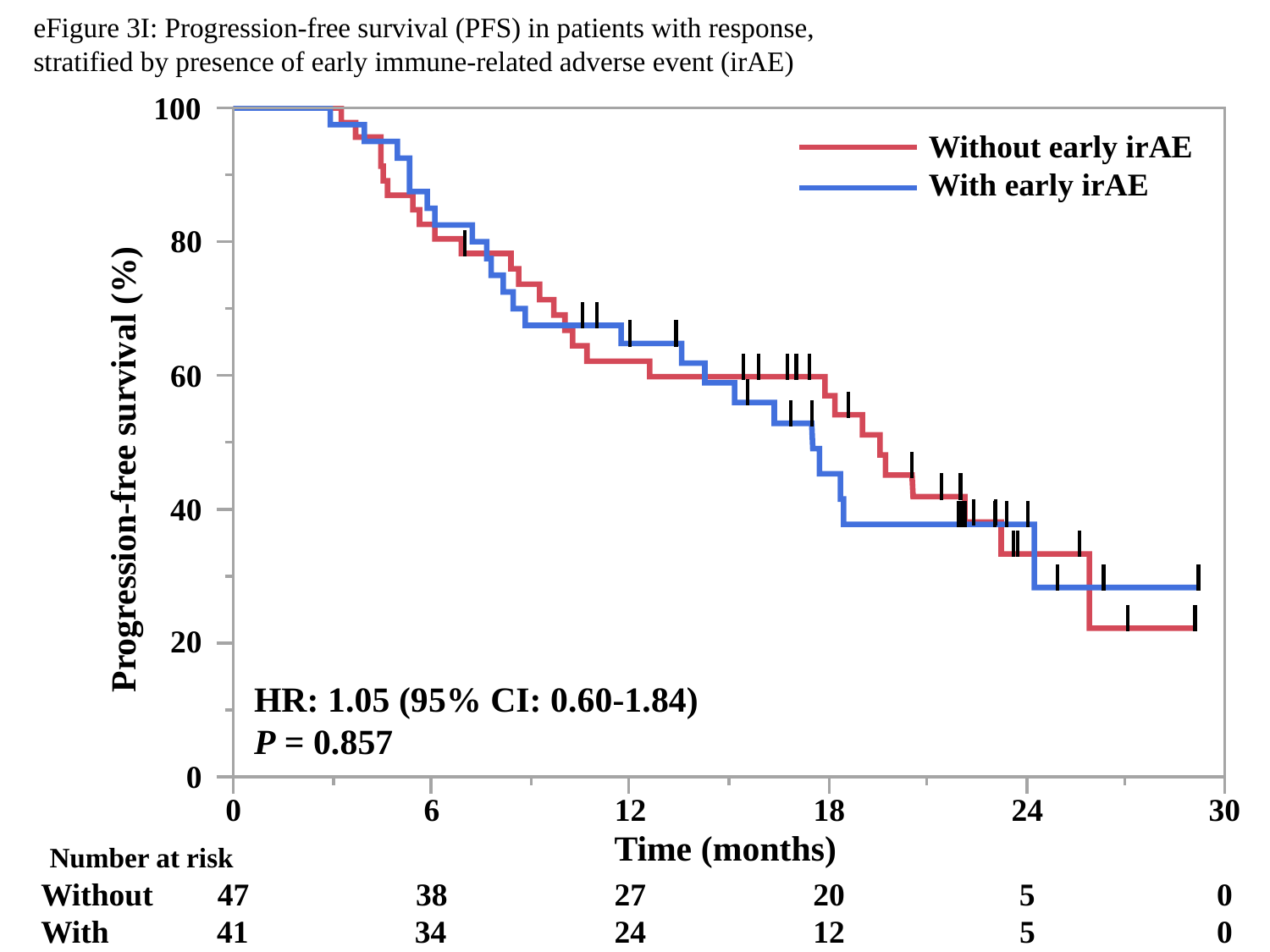

eFigure 3I: Progression-free survival (PFS) in patients with response,
stratified by presence of early immune-related adverse event (irAE)
100
Without early irAE
With early irAE
Progression-free survival (%)
80
60
40
20
HR: 1.05 (95% CI: 0.60-1.84)
P = 0.857
0
0
6
12
18
24
30
Time (months)
Number at risk
Without
47
38
27
20
5
0
With
41
34
24
12
5
0

## Slide 13
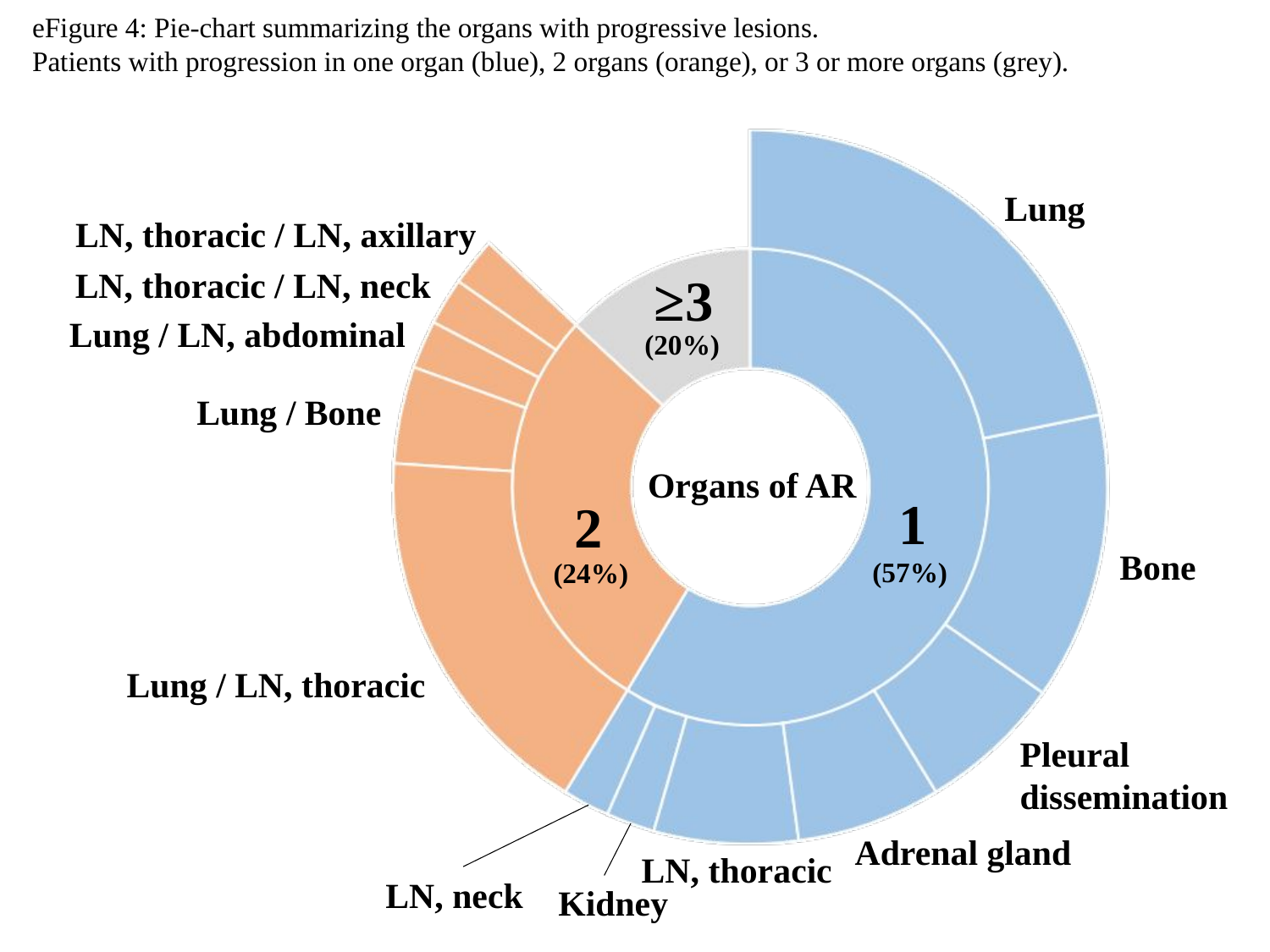

eFigure 4: Pie-chart summarizing the organs with progressive lesions.
Patients with progression in one organ (blue), 2 organs (orange), or 3 or more organs (grey).
Lung
LN, thoracic / LN, axillary
LN, thoracic / LN, neck
≥3
Lung / LN, abdominal
(20%)
Lung / Bone
Organs of AR
1
2
Bone
(57%)
(24%)
Lung / LN, thoracic
Pleural dissemination
Adrenal gland
LN, thoracic
LN, neck
Kidney

## Slide 14
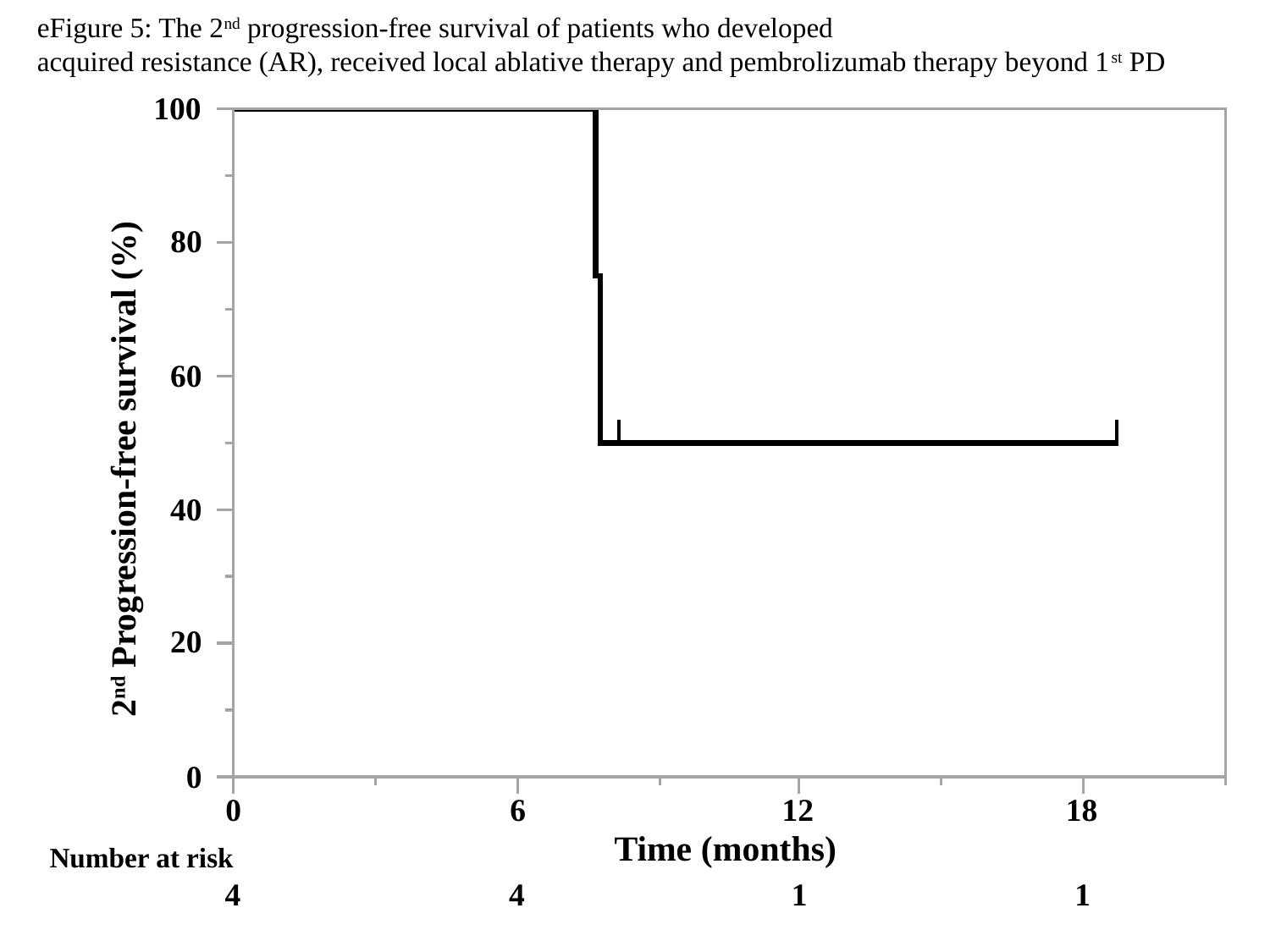

eFigure 5: The 2nd progression-free survival of patients who developed
acquired resistance (AR), received local ablative therapy and pembrolizumab therapy beyond 1st PD
100
2nd Progression-free survival (%)
80
60
40
20
0
0
6
12
18
Time (months)
Number at risk
4
4
1
1
