# Supplementary material for: Clinical factors associated with shorter durable response, and patterns of acquired resistance to first-line pembrolizumab monotherapy in PD-L1-positive non-small-cell lung cancer patients: a retrospective multicenter study
Source: BMC Cancer. 2021 Apr 1;21:346. doi: 10.1186/s12885-021-08048-4 (PMC8017679; doi:10.1186/s12885-021-08048-4)
Supplement: Supplementary file 3 — Additional file 3. [file 12885_2021_8048_MOESM3_ESM.docx]

**Supplementary file 1**

**Ethics Approval and consent to participate**

This retrospective study was approved by the Institutional Review Board in Osaka International Cancer Institute (approval No.1802199367), the Clinical Research Review Committee in Kobe City Medical Center West Hospital (approval No.17-019), the Research Ethics Review Committee in Kobe City Medical Center General Hospital (approval No. zn180402), the Clinical Research Review Committee in National Hospital Organization Kinki-Chuo Chest Medical Center (approval No.631), the Medical Research Ethics Committee in Osaka Habikino Medical Center (approval No.892), the Ethics Committee in Hyogo Prefectural Amagasaki General Medical Center (approval No.29-161), the Medical ethics committee in Kurashiki Central Hospital (approval No.2825), the Clinical Research Review Committee in National Hospital Organization Himeji Medical Center (approval No.29-41), the Clinical Research Review Committee in National Hospital Organization Osaka Toneyama Medical Center (approval No. TNH-20180015), the Clinical Research Review Committee in Osaka General Medical Center (approval No. 30-S03-005), and the Research Ethics Committee in Itami City Hospital (approval No.295-1).
